# A retrospective analysis of surgical techniques and outcomes of hydatid disease in Wasit, Iraq

**DOI:** 10.25122/jml-2021-0093

**Published:** 2022-03

**Authors:** Layth Qassid Al Harbawi, Naseer Kadhim Jawad, Kadhim Jawad AL-Dhahiry, Kasim Sakran Abass

**Affiliations:** 1. Department of Anesthesiology, College of Medical Technology, AL-Kitab University, Kirkuk, Iraq; 2. Department of Surgery, College of Medicine, University of Nineveh, Nineveh, Iraq; 3. AL-Karama Teaching Hospital, College of Medicine, University of Wasit, Wasit, Iraq; 4. Department of Pharmacology and Toxicology, College of Pharmacy, University of Kirkuk, Kirkuk, Iraq

**Keywords:** hydatid cyst, hepatic, surgical removal, intraoperative and postoperative complications

## Abstract

Echinococcosis is a parasitic infestation with high prevalence in Iraq. Surgical treatment remains the standard gold method for treating this disease. The selection of surgical approach depends on the general condition of the patient and characters of the cyst, *e.g.*, size, location, number of cysts, intraoperative findings, and complications such as adhesion, bile leakage, and bleeding. Our study aimed (1) to summarize the most common surgical approaches for treating liver hydatid cyst (HC) in our locality, and (2) to highlight common intraoperative and postoperative complications and the duration of hospital stay. We analyzed the clinical data of 42 patients operated for liver HC. We found that the highest incidence rate of HC was anatomically in the right hepatic lobe with or without synchronous cysts in other organs. The most frequent type of surgery was partial pericystectomy with external tube drainage (ETD) or simple endocystectomy with omentoplasty and ETD. The most important intraoperative finding was cystic-biliary communication. The majority of patients had uneventful postoperative recovery. There is no standardized surgical procedure for hepatic HC. The surgical technique should be modified according to the cyst size, anatomic location of cyst/cysts, number of cysts, cystobiliary communications, cystic infection, and the presence of extrahepatic hydatid cyst or cysts. The surgeon’s experience plays a vital role in selecting the surgical technique for hepatic hydatid cystectomy.

## INTRODUCTION

Hydatid cyst (HC), also known as hydatidosis, echinococcosis, or liver full of water, is a potentially serious disease. Human infestation occurs via fecal-oral transmission of parasitic ova from dog feces or other canines [1–3]. Upon ingestion, the ova hatch in the human intestine into six-hooked oncospheres, which penetrate the intestinal wall and migrate into various tissues, particularly the liver (50–90%), lungs (10–15%), or any other organ [4–6]. In these organs, the oncospheres develop into a thick wall cyst filled with protoscolices and daughter cysts. Patients with HC remain asymptomatic until the size of the cyst becomes large enough to cause discomfort or rupture of the cyst into the adjacent tissues. The cystic rupture results in a spread of scolices, and a highly antigenic hydatid fluid triggers mild to severe widespread hypersensitivity reactions. Non-surgical approaches have been used to treat HC, such as percutaneous aspiration, injection of chemicals, and reaspiration (PAIR). Nevertheless, surgical excision remains the standard gold treatment and can bring about a complete cure [7–9].

The key aspect of HC treatment is to evacuate the cysts and their contents without leakage of the hydatid fluid into the operative field to avoid other complications. The decision for surgical techniques depends on the condition of the patient, the status of the cyst before exploration, intraoperative findings, and the surgeon’s experience. Preoperative imaging is essential to determine the location, size, number of cysts, accessibility, and the presence or absence of concomitant cysts outside the liver. Intraoperatively, the surgical technique can be modified according to nearby unexpected complications or findings.

This study summarizes the most frequent surgical techniques advocated for surgical treatment of hepatic HC in our locality. Moreover, it highlights the most common intraoperative and postoperative complications and the duration of hospital stay.

## MATERIAL AND METHODS

We performed a retrospective analysis of the medical records of 42 patients who presented to AL-Karama teaching hospital, Wasit, Iraq, from July 2011 to November 2018. These patients presented with multiple or large hepatic HC and were treated by open surgical technique rather than laparoscopy. The following data were extracted: age, gender, residence urban/rural, signs and symptoms, radiological findings, intraoperative findings, intra and postoperative complications, duration of hospital stay, and the follow-up period. The diagnosis was confirmed with abdominal ultrasonography and spiral CT scans. These radiologic modalities were used to evaluate the cyst size, location, extent, and classification according to the World Health Organization (WHO) [10].

### The surgical techniques

Open surgery rather than laparoscopy was the preferred surgical technique for patients with large or multiple hepatic hydatid cysts and previous upper abdominal surgery. In 62% of the study patients, the Clavien system of surgical removal of HC was used. First, aspiration of some of the hydatid cyst fluid with a fine 5-milliliter syringe needle was performed to verify if the fluid was bile-stained. Then, some of the hydatid fluid was sucked out with a closed vacuum system using a wide pore needle. Afterwards, scolicidal solution was injected into the cyst, either povidone-iodine 10% if the hydatid fluid was not bile-stained or hypertonic saline 10% if the hydatid fluid was bile-stained. The scolicidal agent was left in the hydatid cyst for 8–12 minutes to kill the parasites and inactivate the germinal layer. Next, the hydatid cyst fluid and daughter cysts were removed using a wide pore sucker and scoop. Then, the endocyst was removed, and the residual cavity was checked for any bile leak. If no leak was found, the surgeon proceeded to partial pericystectomy, leaving parts of the cyst wall closely adherent to local blood vessels and biliary passages to avoid biliary injury and bleeding. Management of the residual cavity was according to its size and the presence of cystobiliary communication. If the residual cavity was clean, of moderate size, without bile leak, and located in the anterior segments of the liver, it was filled with an axial omental flap (omentoplasty) with external tube drainage (ETD) of Morrison’s pouch. If the residual cavity contained a bile leak, it was treated with omentoplasty and ETD inside the cavity after suturing the bile leak with 3/0 or 4/0 prolene. If the residual cavity was too large to be filled with an omental flap or was located in the posterior segments of the liver, it was treated by partial pericystectomy.

## RESULTS

### Characteristics of participants

Forty-two patients were included in this study, with n=28 females (66.6%) and n=14 males (33.3%). The mean age was 40 years (range 10–80 years). 59% of the study patients were from rural areas. The most commonly reported clinical manifestations were right hypochondrial pain and epigastric pain with or without palpable liver. Additionally, some patients presented with vomiting and attacks of fever, rigor, and jaundice.

### Cyst characteristics

The numbers of cysts shown by abdomen ultrasonography were: 35 patients had a single cyst (83.3%), one patient (2.38%) had two cysts, 2 patients (4.76%) had 3 cysts, one patient (2.38%) had 4 cysts, 2 patients (4.76%) had 7 cysts and one patient (2.38%) had 10 cysts. The size of the cysts ranged from 7 to 25 centimeters (cm), and 80% of them were located in the right hepatic lobe. Twenty-eight (66.7%) cysts were located solely in the liver, whereas, in the remaining patients, there were concomitant cysts in the lung, spleen, pancreas and/or peritoneum. Furthermore, patients’ data showed that 37 (88%) of the hepatic hydatid cysts were primary ones, while in 5 patients (12%), the cysts were recurrent (Table 1).

**Table 1.** Demographics and key characteristics of hydatid disease in the study.

Study Variables		Number	%
**Sex**	**Female**	28	66.7
	**Male**	14	33.3
**Age groups/year**	**10–20**	2	4.8
	**21–30**	7	16.7
	**31–40**	16	38
	**41–50**	11	26
	**51–60**	3	7
	**61–70**	2	4.8
	**>70**	1	2.4
**Location**	**Urban**	17	40.5
	**Rural**	25	59.5
**Type of cyst**	**Primary**	37	88
	**Recurrent**	5	12
**Location of the cyst**	**Liver only**	28	66.7
	**Liver+lung**	6	14
	**Liver+spleen**	2	4.8
	**Liver+pancreas**	1	2.4
	**Liver+peritoneum**	5	12

About 96% of these cysts were Gharbi (G) type I and Gharbi type III according to WHO classification (Table 2). All patients received Albendazole (10 m/kg) for 4 weeks preoperatively and continued for about 3 months after surgery.

**Table 2.** Frequency of liver hydatid cysts according to Gharbi WHO Classification [10].

Type	Description	Frequency
**I**	Pure univesicular fluid accumulation with a “double line” sign	12 (28.5%)
**II**	Fluid accumulation with detached membrane (water Lily) sign	2 (4.8%)
**III**	Fluid accumulation with septa and/or daughter cysts (Rosette-like)	24 (57%)
**IV**	Uniformly echogenic cyst pattern	3 (7%)
**V**	Cyst with thick calcified borders	1 (2.4%)

### Surgical Technique Results

Twenty-six patients (62%) presented with hepatic HC types GII, GIII, and GIV, were treated with partial pericystectomy after sterilizing the cyst with povidone-iodine (if hydatid fluid was crystal clear) or hypertonic saline 10% (if hydatid fluid was bile stained), followed by insertion of ETD with or without omentoplasty. Other cysts were treated with simple endocystectomy and deroofing (marsupialization) ETD of the residual cavity and Morrison’s pouch ETD (Table 3).

**Table 3.** Types of surgical procedures performed, intraoperative findings, intraoperative complications, and postoperative complications.

Variables	Descriptions	Number	%
**Type of operative procedure**	Pericystectomy + ETD	26	62
	Endocystectomy and Marsupialization ETD and/or Omentoplasty	16	38
**Intraoperative findings and complications**	Cysto-biliary communication	6	14.3
	Bleeding	2	4.8
	Infected cyst	2	4.8
	None	32	76
**Postoperative complications**	Biliary leakage	6	14.3
	Biliary fistula	6	14.3
	Others (fever, chest infection, vomiting...)	30	71.4

Cholecystectomy was performed for 8 patients, in 6 of them, the gallbladders were part of the cyst wall, while the remaining 2 patients had chronic calculous cholecystitis. Two patients also had splenectomy for concomitant splenic HC. One female patient presented with obstructive jaundice due to intrabiliary rupture of HC in the liver, obstructing extrahepatic bile by daughter cysts. Endoscopic retrograde cholangiopancreatography (ERCP) failed to remove the daughter cysts from the extrahepatic bile ducts. This patient was treated with open hydatid cystectomy to explore the common bile duct and choledochodoudenostomy.

### Intraoperative Findings and Complications

Communication between the cyst and the biliary tree was found in 6 patients, bleeding that spontaneously stopped occurred in 2 patients, and infection occurred in 2 patients (Table 3). Neither anaphylactic shock nor intraoperative or postoperative mortality was recorded.

### Postoperative Complications

Twelve patients (28.57%) had postoperative bile leakage that spontaneously stopped during one to 12 weeks. These patients had cystobiliary communications (bile-stained hydatid fluid). Most patients (71.4%) had non-specific complications such as fever, pain, vomiting, mild jaundice, and chest or wound infection that conservatively improved (Table 3). Postoperative hospital stay ranged between 3–7 days.

## DISCUSSION

Hydatid disease is a parasitic disease (larva) caused by a tapeworm called Echinococcus granulosus, which can be transmitted to man either directly by contact with dogs or indirectly by ingesting foods or water contaminated with the excreta of infected animals.

In the current study, 66.7% of the patients were females, and 33.3% were males. Furthermore, 59.5% of patients lived in rural areas, while 40.5% lived in urban areas. Given that more patients live in rural areas, they could be in contact with herbivorous animals. Furthermore, many wild dogs feed on these animal products illegally slaughtered out of slaughterhouses. This finding agrees with the results reported by other studies [11–14].

Treatment of hepatic HC can be performed by surgical and non-surgical methods depending on the condition of the patient and the status of the cyst. In the current study, hepatic HCs of ≥7 cm in diameters were treated by open surgery, which is still considered the gold standard for treating hepatic HC. All patients received Albendazole 10 mg/kg for 4 weeks before surgery and continued after that for 3 months to decrease the risk of recurrence. Furthermore, type GI and GII cysts can be successfully treated by PAIR (percutaneous aspiration injection re-aspiration [15] or by laparoscopic treatment [16]. Conservative surgery usually carries the risks of recurrence and infection compared to the pericystectomy method [17–18]. Such cysts can be managed by other procedures like PAIR (percutaneous aspiration, scolicidal injection, and re-aspiration), thermal ablation, chemotherapy, particularly in unfit patients [7, 8, 19–21].

In this study, cystobiliary communications were detected intraoperatively in 6 patients (14.28%) that developed postoperatively into biliary fistulae. This complication was also reported in many studies, particularly in GIII and GIV cysts [18–20, 22]. In addition, cholecystectomy was performed in 8 patients (19%) due to the gall bladder being part of the HC wall or chronic calculous cholecystitis.

Profuse bleeding occurred in two patients. The first patient complained of a third recurrence of hepatic HC with dense adhesions of the cyst and liver to the surrounding abdominal structures. The second patient had pancreatic bleeding during the removal of concomitant pancreatic HC. In both patients, the bleeding was controlled intraoperatively. In 71.43% of operated patients, omentoplasty was applied to close the residual cavity, ending in a smooth postoperative period without bile leakage or infection. Omentoplasty *vs.* tube drainage can be used even for patients treated conservatively to decrease postoperative complications and hospital stay [19, 23–25].

**Figure 1.**
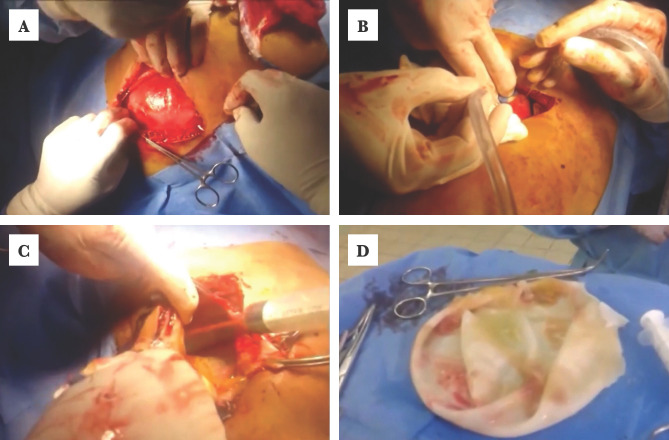
A – Exploration of the hydatid cyst; B – Suction of hydatid fluid by a closed vacuum system; C – Deroofing of the dome of the hydatid cyst; D – Hydatid cyst wall measuring 25 cm in diameter (it contained more than 4 liters of hydatid fluid).

One female patient of 38 years old presented with a large abdominal mass; ultrasonography revealed a cyst of 25 centimeters (cm) occupying most of the right hepatic lobe. Intraoperatively, the cyst had a thick wall, and the bile was not stained (Figure 1 A–D).

A partial pericystectomy was performed, and an ETD was left in the residual cavity. Postoperative recovery was uneventful. Another patient was a 10-year-old boy who presented with progressive abdominal painful distention. Abdominal ultrasonography and CT scan revealed multiple abdominal HCs. On exploration, there were 10 cysts 4–5 cm in diameter, all GI type. Those situated in the peritoneum were removed by complete pericystectomy, while those embedded deep in the liver were dealt by simple endocystectomy and marsupialization of the residual cavity. One tube drain was placed in the right subphrenic space, and another tube was put into Morrison’s pouch.

Postoperative periods of the study patients were generally uneventful, and hospital stay ranged from 3–7 days depending on the difficulty of the operation, the general condition of the patient, and the presence of any post complications [26, 27].

## CONCLUSIONS

There are no standardized surgical procedures for HC in the liver. The surgical technique can be tailored according to the cystic size, the number of cysts, anatomic location of the cysts, presence of cystobiliary communications, intra-abdominal extrahepatic hydatid, and the surgeon’s experience. The surgeon’s experience plays a vital role in determining the surgical technique for hepatic hydatid cystectomy. Good sterilization of the cyst contents with a safe scolicidal and meticulous removal of the cystic contents are essential operative steps to prevent recurrence and secondary bacterial infection of the residual cavity. Moreover, obliterating the residual cavity or partial pericystectomy reduces the cyst cavity infection risk. Future randomized and prospective studies are necessary to validate present findings.

## ACKNOWLEDGMENTS

### Conflict of Interests

The authors declare no conflict of interest.

### Ethical approval

The study was approved by the Audit Committee of the Al-Kitab University Trust (approval ID: 113).

### Personal thanks

The author wishes to thank the AL-Karama teaching hospital staff from Wasit Governorate.

### Authorship

LA contributed to data collection and writing the article. KN and KD designed the methodology, and KA reviewed the article.
